# Molecular Genetic Characterization of the Diet of Limestone and Rainforest Langurs

**DOI:** 10.1002/ece3.73892

**Published:** 2026-06-30

**Authors:** N. Van Truong, D. Groth, L. Hallmaier‐Wacker, S. Knauf, A. Poehlein, L. Duc Minh, T. Nadler, L. Zhang, X. Wang, L. T. Anh, M. Li, C. Roos, M. Hofreiter

**Affiliations:** ^1^ Evolutionary Adaptive Genomics, Institute of Biochemistry and Biology, Faculty of Science University of Potsdam Potsdam Germany; ^2^ Primate Genetics Laboratory German Primate Center, Leibniz Institute for Primate Research Göttingen Germany; ^3^ Central Institute for Natural Resources and Environmental Studies Vietnam National University Hanoi Vietnam; ^4^ Bioinformatics, Institute of Biochemistry and Biology, Department of Science University of Potsdam Potsdam Germany; ^5^ Institute of International Animal Health/One Health, Friedrich‐Loeffler‐Institute Federal Research Institute for Animal Health Greifswald ‐ Insel Riems Germany; ^6^ Faculty of Veterinary Medicine Justus Liebig University Giessen Germany; ^7^ Department of Genomic and Applied Microbiology Georg‐August University of Göttingen, Institute of Microbiology and Genetics Göttingen Germany; ^8^ Cuc Phuong Commune Ninh Binh Province Vietnam; ^9^ CAS Key Laboratory of Animal Ecology and Conservation Biology, Institute of Zoology Chinese Academy of Sciences Beijing China; ^10^ Mien Trung Institute for Scientific Research Vietnam National Museum of Nature Hue Vietnam; ^11^ Center for Excellence in Animal Evolution and Genetics Chinese Academy of Sciences Kunming China; ^12^ Gene Bank of Primates German Primate Center, Leibniz Institute for Primate Research Göttingen Germany

**Keywords:** adaptive evolution, folivory, internal transcribed spacer (ITS), limestone langur, nonhuman primates, rainforest langur

## Abstract

The diet of wild nonhuman primates is still largely unknown, but advances in molecular techniques using DNA metabarcoding allow obtaining such information much faster and in more detail than until recently. The genus *Trachypithecus* is the most speciose and most broadly distributed genus among Asian colobines, a group of highly folivorous primates. The genus contains four groups, of which three occur in rainforest habitats while the fourth, the *T. francoisi* group, is restricted to limestone habitat. We examined whether dietary diversity and composition vary among species and between ecological groups (limestone vs. rainforest), reflecting habitat‐associated ecological differences. To characterize the food plant composition of langurs, we employed a metabarcoding approach and analyzed monthly samples (*n* = 419) collected over a 1‐year period from *T. crepusculus* and *T. germaini* , members of the *T. obscurus* and *T. cristatus* groups, respectively, and *T. delacouri* and *T. hatinhensis* , both members of the *T. francoisi* group. By identifying plant taxa primarily to the genus level, we found that langur species consume a large number of different plants, with diets comprising 122–129 plant genera across 59–73 families. Statistical comparisons revealed significant differences between rainforest and limestone habitats, with higher abundance in the rainforest species (*p* < 0.001) and a large effect size (ε^2^ ≥ 0.8). Despite these differences, dietary composition showed substantial overlap between rainforest and limestone langurs. The four species also differed in their reaction to seasonal changes, as some species consumed different plants during the dry and wet seasons, whereas others exhibited relatively stable plant consumption over the year. Despite the pronounced differences between rainforest and limestone habitats, ecological groups were not clearly distinct. Overall, our results suggest that both phylogenetic relatedness and habitat influence diet. This study improves our understanding of langur ecology and dietary composition at both plant genus and family levels.

## Graphical abstract

Using DNA metabarcoding of 419 fecal samples collected over 1 year, we characterized and compared the diets of four *Trachypithecus* species from rainforest and limestone habitats in Vietnam. Langurs consumed a remarkably high diversity of plants (122–129 genera across 59–73 families), with clear interspecific differences in dietary composition and seasonal responses, but no distinct separation between rainforest and limestone species. This study provides a comprehensive dietary inventory of *T. crepusculus* in Vietnam and advances understanding of ecological variation within the genus.

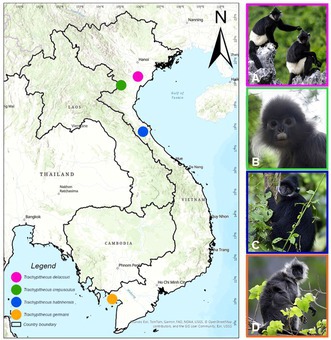

## 1 Introduction

Nonhuman primates play an important role in ecosystems, form a key component of tropical biodiversity, and help to understand human evolution (Wallace et al. 2011; Mariño 2016; Marshall and Wich 2016). Dietary adaptations are an important component of a species' ecological niche and influence both current and potential future distributions. Thus, knowledge about dietary adaptations helps to understand the ecology and evolutionary history of species (Hemingway and Bynum 2005; Marshall and Wrangham 2007; Clink et al. 2017), and also how species are distributed and affected by habitat changes. Moreover, exploring their diets can be critical to understand their impact on plant communities and ecosystem function (Chapman 1995; Garber and Lambert 1998; Chapman and Russo 2007; Andresen et al. 2018; Nevo et al. 2018).

Diet composition is traditionally investigated either via direct observation of feeding animals, or by morphological analysis of fecal samples (Moreno‐Black 1978; McInnis et al. 1983; Schneider et al. 2010; Koirala et al. 2016; Thiry et al. 2025). However, these methods are limited in terms of human expertise and resources as well as costs and time required, and there are also various factors that can bias the results (Altmann 1991; Rothman et al. 2013). As an alternative, metabarcoding approaches can be used for dietary characterization from feces (Gonzalez et al. 2009; Quéméré et al. 2013; Bell et al. 2016; Kress 2017; de Sousa et al. 2019; Fonseca et al. 2022; Tercel et al. 2022). Often, a larger number of plants and therefore more detailed information on animal diets can be detected using DNA barcoding techniques in combination with next‐generation sequencing compared to traditional methods (Gu et al. 2013; Bell et al. 2016; Jeanniard‐du‐Dot et al. 2017; Granquist et al. 2018; de Sousa et al. 2019; Harper et al. 2020; Thiry et al. 2025).

Colobine monkeys (family Cercopithecidae, subfamily Colobinae) occur in Africa and Asia and are the primate group most specialized on a folivorous diet. Among Asian colobines, the genus *Trachypithecus* is the most speciose with a total of 22 species (Mittermeier et al. 2013; Roos et al. 2014, 2020; Roos and Zinner 2022; Roos 2022). It is also the most widely distributed Asian colobine genus, ranging from Bhutan and Assam in the West to southern China and Vietnam in the East, and South to Sundaland (Sumatra, Borneo, and Java) (Brandon‐Jones et al. 2004; Mittermeier et al. 2013; Roos et al. 2014, 2020; Roos 2022). Based on similarities in phenotype, behavior, ecology, and genetics, the 22 species are divided into four species groups (Mittermeier et al. 2013; Roos et al. 2014, 2020; Roos and Zinner 2022; Roos 2022). Three of them, the *T. obscurus* , *T. cristatus* and *T. pileatus* groups, are mainly found in rainforest habitats, while the fourth, the *T. francoisi* group, is restricted to karst habitats in Laos, southern China, and Vietnam (Mittermeier et al. 2013; Roos et al. 2014, 2020; Roos 2022; Roos and Zinner 2022). These karst habitats, known for their unique landscapes and rich biodiversity, are also covered by rainforest, but with a distinct plant species composition compared to typical (i.e., non‐karstic) rainforest habitats (Clements et al. 2006; Luo et al. 2016; Wang et al. 2019). The seven species of the *T. francoisi* group, sometimes also collectively called limestone langurs, are unique among primates (Mittermeier et al. 2013; Roos et al. 2014, 2020; Roos and Zinner 2022; Roos 2022), as they are known to sleep in caves or under rock shelters, can easily walk, climb, and leap on sharp rocks (Osterholz et al. 2008; Workman 2010), and exhibit genomic adaptations to naturally high calcium intake in form of drinking water and food plants (Liu et al. 2020, 2025; Zhang et al. 2024).

The aim of this study was to characterize the feeding ecology of four *Trachypithecus* species, representing limestone langurs ( *T. delacouri* and *T. hatinhensis* ) and rainforest langurs ( *T. crepusculus* and *T. germaini* ), whereby the investigated population of *T. germaini* occurs in limestone habitat. We used a DNA metabarcoding approach to examine whether dietary composition varies between limestone and rainforest associated langur species, noting that habitat type is partially confounded with species identity and geographic distribution in this study. Dietary data were generated by amplifying and sequencing the internal transcribed spacer 2 (ITS2) region of nuclear ribosomal DNA extracted from fecal samples. Simultaneously, we evaluated potential differences in dietary composition over the year by analyzing monthly collected fecal samples for each species.

## 2 Methods

### 2.1 Sample Collection

**FIGURE 1:**
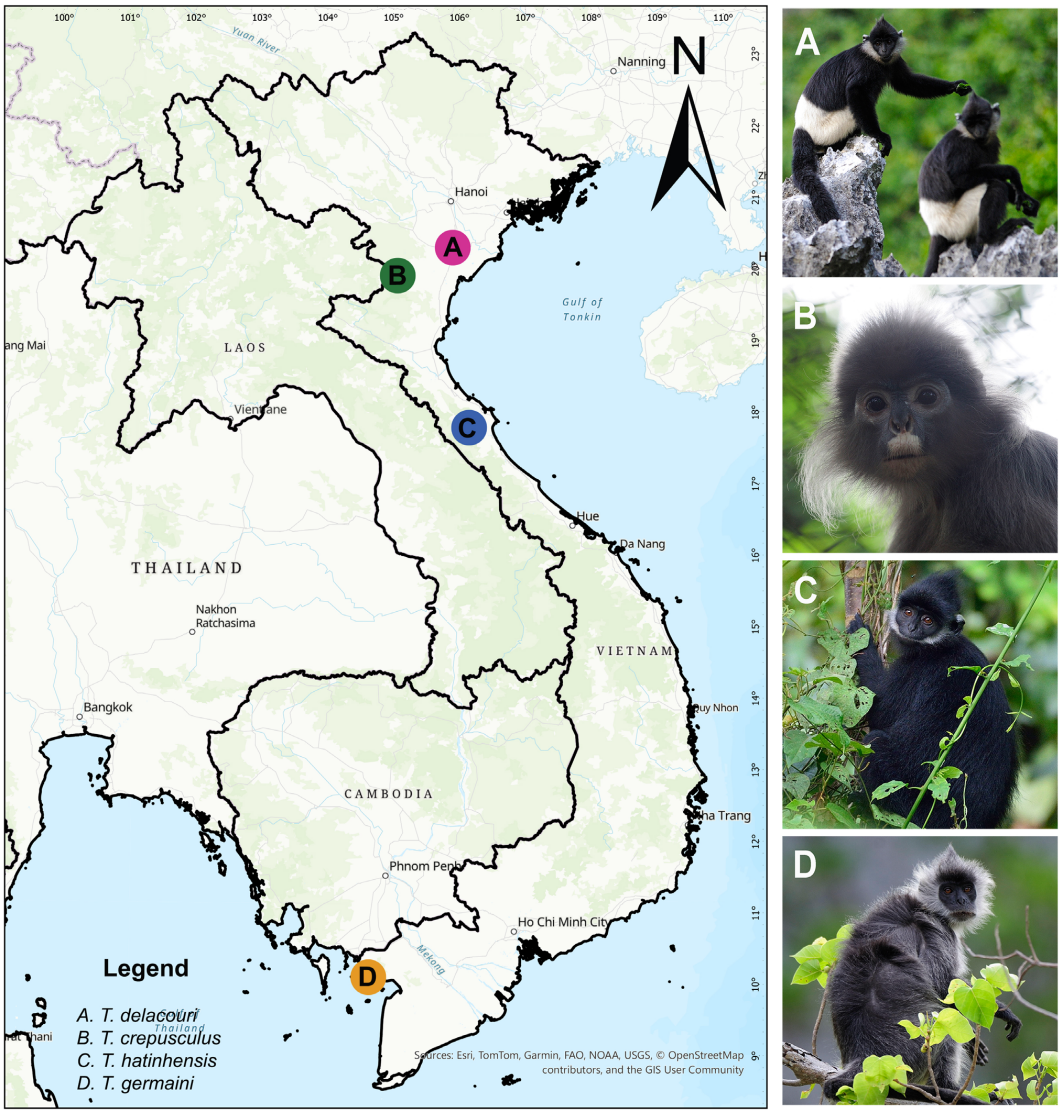
Study sites in Vietnam and photographs of the four study species (A) Delacour's langur—Tdel ( *T. delacouri* ) in Van Long Nature Reserve, Ninh Binh Province, (B) Indochinese gray langur—Tcre ( *T. crepusculus* ) in Xuan Lien National Park, Thanh Hoa Province, (C) Hatinh langur—That ( *T. hatinhensis* ) in Tuyen Phu Forest, Quang Tri Province, and (D) Indochinese silvered langur—Tger ( *T. germaini* ) in Chua Hang Karst Forest, An Giang Province (Photographs: Nguyen Van Truong). The map was generated with ESRI ArcGIS 10.2 (ESRI 2013, Redlands, CA, USA; https://www.esri.com). Photographs were retouched using Adobe Photoshop 2025 (Adobe Inc. 2025, San Jose, CA, USA).

Fecal samples were collected noninvasively from wild populations of four *Trachypithecus* species in four protected areas of Vietnam, three of which are in the north while one is in the south of the country (Table 1, Figure 1). The four species investigated represent limestone langurs ( *T. delacouri* and *T. hatinhensis* ) and rainforest langurs ( *T. crepusculus* and *T. germaini* ); however, the study population of *T. germaini* lives in limestone habitat (Figure 1, Table 1). *Trachypithecus delacouri* was specifically sampled in Van Long Nature Reserve (Ninh Binh Province; evergreen and some deciduous forests on limestone), *T. hatinhensis* in Tuyen Phu Forest (Quang Tri Province; evergreen tropical forest on limestone), *T. germaini* in Chua Hang Karst Forest (An Giang Province; evergreen monsoon forest on limestone), and *T. crepusculus* in Xuan Lien National Park (Thanh Hoa Province; broadleaf evergreen forest) (Table 1, Figure 1). Wet and dry seasons were defined based on regional climate patterns (Table 1, Table S1). Seasonal classification was therefore applied separately for each site to account for spatial variation in rainfall across Vietnam (Table S1).

**TABLE 1:** Information about study species, their species group classification (LL: Limestone langur; RL: Rainforest langur), habitat classification (LH: Limestone habitat; RH: Rainforest habitat), details about sampling location, period of dry, and wet season, and number of collected samples.

Species	Species group classification	Habitat	Location	Latitude longitude	Dry season	Wet season	Sample nr.
*T. delacouri*	LL	LH	Van Long Nature Reserve, Ninh Binh Province	20°23′05″ N 105°52′31″ E	Nov‐April	May‐Oct	100
*T. hatinhensis*	LL	LH	Tuyen Phu Forest, Quang Tri Province	17°52′25″ N 106°07′17″ E	Nov‐Jun	Jul‐Oct	116
*T. germaini*	RL	LH	Chua Hang Karst Forest, An Giang Province	10°10′08″ N 104°37′32″ E	Nov‐May	Jun‐Oct	122
*T. crepusculus*	RL	RH	Xuan Lien National Park, Thanh Hoa Province	19°56′32″ N 105°01′48″ E	Nov‐Apr	May‐Oct	81

All field assistants were properly trained to monitor and observe the animals and on how to collect the samples. In total, 419 fecal samples were collected over a 12‐month period between January 2019 and March 2020 (Table 1, Table S1). At each site, we aimed to collect 10 fecal samples per species per month (Table 1). For each species, monthly samples were collected from the same social group, which contained, depending on species, 15–20 individuals. By observing animals during defecation, the risk of repeatedly sampling the same individual was minimal, but individual identity over the months was not traceable. Approximately 3 g of fresh fecal material was collected and preserved in 7 mL of RNA later. Samples were maintained at 4°C for at least 24 h and then transported on dry ice for long‐term storage at −20°C.

Fecal samples were collected without disturbing the animals. This study followed the International Primatological Society Ethical Guidelines for the Use of Nonhuman Primates in Research (Riley et al. 2014), as well as the ethical guideline of the German Primate Center (Goettingen, Germany) and the University of Potsdam (Germany). All necessary permits for fieldwork were obtained from relevant Vietnamese authorities, including national parks, nature reserves, and the management boards of special‐use forests. Approval to export samples was granted by the Ministry of Natural Resources and Environment (MONRE) (No: 1247/QĐ‐BTNMT) on 04 June 2020, and CITES permit (No: 200756/CITES‐VN) on 25 November 2020.

### 2.2 DNA Extraction, Amplification and Sequencing

DNA from fecal samples was extracted with the NucleoSpin DNA Stool Kit (Macherey‐Nagel, Düren, Germany) following the manufacturer's instructions. Extraction procedures included negative controls to monitor potential contamination. Purified DNA was quantified with a Qubit 2.0 fluorometer (Thermo Fisher Scientific, Waltham, MA, USA) and stored at −20°C until further processing. The ITS2 DNA barcode was amplified by polymerase chain reaction (PCR) using the universal UniPlant primer pair, UniPlantF (5′‐TGTGAATTGCARRATYCMG‐3′) and UniPlantR (5′‐CCCGHYTGAYYTGRGGTCDC‐3′) (Moorhouse‐Gann et al. 2018). To simplify library preparation, we added adapter sequences to the 5′‐ends of the primers (forward overhang: 5′‐TCGTCGGCAGCGTCAGATGTGTATAAGAGACAG‐[locus‐specific sequence]‐3′; reverse overhang: 5′‐GTCTCGTGGGCTCGGAGATGTGTATAAGAGACAG‐[locus‐specific sequence]‐3′).

All PCRs, including negative controls, were performed in triplicates in a total volume of 25 μL, containing 12.5 μL of Platinum II Hot‐Start PCR Master Mix, 5.0 μL of Platinum GC enhancer, 0.5 μL of both primers (0.2 μM each), 5.5 μL of nuclease‐free water, and 1 μL (ca. 50 ng) template DNA. The thermal cycling conditions consisted of an initial denaturation at 94°C for 3 min, followed by 40 cycles of 94°C for 45 s, 56°C for 1 min, and 72°C for 90 s, and a final extension at 72°C for 10 min. PCR products were visualized on 2% agarose gels.

PCR products were then purified using the Solid‐Phase Reversible Immobilization (SPRI) selection method with the SPRIselect bead‐based reagent (Beckman Coulter, Brea, CA, USA) using a bead to PCR product ratio of 0.7. PCR performance and cleanup were checked on a Bioanalyzer 2100 or TapeStation 2200. Triplicates were pooled in equimolar amounts (10 nM) and then subjected to indexing PCR. PCR products were used to attach indices and Illumina sequencing adapters using the Nextera XT Index kit (Illumina, San Diego, CA). Index PCR was performed using 5 μL of template PCR product, 2.5 μL of each index primer, 12.5 μL of 2× KAPA HiFi HotStart ReadyMix and 2.5 μL PCR grade water. The thermal cycling scheme used was as follows: 95°C for 3 min, 8 cycles of 30 s at 95°C, 30 s at 55°C and 30 s at 72°C and a final extension at 72°C for 5 min. Quantification of the products was performed using the Quant‐iT dsDNA HS assay kit and a Qubit fluorometer (Invitrogen GmbH, Karlsruhe, Germany) following the manufacturer's instructions. MagSi‐NGS^PREP^ Plus Magnetic beads (Steinbrenner Laborsysteme GmbH, Wiesenbach, Germany) were used for purification of the indexed products as recommended by the manufacturer, and normalization was performed using the Janus Automated Workstation from Perkin Elmer (Perkin Elmer, Waltham Massachusetts, USA). Sequencing was conducted on the Illumina MiSeq platform using dual indexing and MiSeq reagent kit v3 (600 cycles) as recommended by the manufacturer.

### 2.3 Bioinformatic Workflow

The raw sequence reads were processed with the Mothur software package v1.46.1 (Schloss et al. 2009), following MiSeqSOP (Kozich et al. 2013). Paired‐end sequences (forward and reverse reads) were merged using the make.contigs command with default settings. In the next step, we filtered our dataset by removing merged reads using the screen.seqs command according to the following criteria: merged reads below 200 bp and above 500 bp and reads with quality score ≤ Q30 were removed. We subsequently also removed duplicate sequences using the unique.seq command and eliminated sequencing artifacts known as chimeras using vsearch (chimera.vsearch command) (Rognes et al. 2016). To prepare the data input for MEGAN (Huson et al. 2007), we initially generated a local BLAST database of the ITS region of all plant species available in GenBank (Madden 2013; Sen et al. 2017). Next, we compared the remaining 9.8 million high‐quality sequences obtained through the previous steps against this database using BLASTn v2.12.0+ (Camacho‐Benavides et al. 2013). Subsequently, we employed the BLAST results as input for MEGAN Community Edition v6.24.4 to determine taxonomic rank using the Lowest Common Ancestor (LCA) algorithm with default parameters (Huson et al. 2007). For subsequent analyses, data obtained from individual fecal samples were combined for each langur species, depending on the analysis either separately for each month or for the full observation period.

### 2.4 Statistical Analysis

Statistical analyses were conducted in R v4.2.3 (R Core Team 2022) using the *vegan, tidyverse*, and *psych* packages (Wickham 2021; Oksanen 2022; Revelle 2022). Alpha diversity indices, including Shannon's diversity (Shannon 1948) and evenness, were calculated using these packages. We also performed a multidimensional scaling (MDS) analysis based on Spearman correlation‐derived distances (D = (1 − ρ)/2) for plant families using the standard R function *cmdscale*. Further, principal component analysis (PCA) was performed using the standard R function *prcomp* on either raw abundance data or *z*‐score–scaled and log_2_‐transformed *z*‐score data. PCA visualization was carried out using the R package *sbi* (https://github.com/mittelmark/sbi). We performed pairwise nonparametric comparisons using Wilcoxon rank‐sum tests to assess differences in taxonomic composition between predefined ecological groupings. Two contrasts were evaluated: (i) *T. crepusculus* (rainforest habitat) versus *T. delacouri* + *T. hatinhensis* + *T. germaini* (all limestone habitat), and (ii) *T. crepusculus* + *T. germaini* (both rainforest langurs) versus *T. delacouri* + *T. hatinhensis* (both limestone langurs). Wilcoxon effect sizes (*r*) were calculated to quantify the magnitude of differences between groups. To reduce noise, analyses were restricted to plant families in which at least one species exceeded 1% relative abundance in at least one comparison group. In addition, we applied the nonparametric Kruskal–Wallis test followed by Dunn's post hoc comparisons, and calculated epsilon‐squared effect sizes to assess differences in diet among the four langur species. Differences between langur species pairs were investigated using analysis of variance (ANOVA), and *p*‐values were adjusted using a Tukey's post hoc test. Beta diversity was calculated using Bray–Curtis dissimilarity (Bray and Curtis 1957) and Unweighted UniFrac distances (Lozupone et al. 2007, 2011). In addition, analysis of molecular variance (AMOVA; Excoffier et al. 1992) was conducted in Mothur to assess dietary differences across all four langur species, between limestone and rainforest groups, and among species pairs at both plant family and genus levels. The data used to generate the figures in this paper are publicly available in the vignette of the R *trachy* package (https://github.com/mittelmark/trachy).

## 3 Results

### 3.1 Data Set

We sequenced a total of 419 samples from wild populations of four *Trachypithecus* species, *T. delacouri* (*n* = 100), *T. hatinhensis* (*n* = 116), *T. germaini* (*n* = 122), and *T. crepusculus* (*n* = 81) (Table 1). We generated a total of 19,029,087 sequencing reads, corresponding to a mean and standard error (SE) of 45,415 ± 1029, a median of 39,409, and a range of 1501–216,738 reads per sample (Table S1). After quality filtering, a total of 9,830,944 reads (52%) from 415 samples remained, with a mean of 23,689 ± 521 SE, a median of 21,169, and a range of 1533–100,064 reads per sample (Table S1).

### 3.2 Habitat Effects on Dietary Composition

**FIGURE 2:**
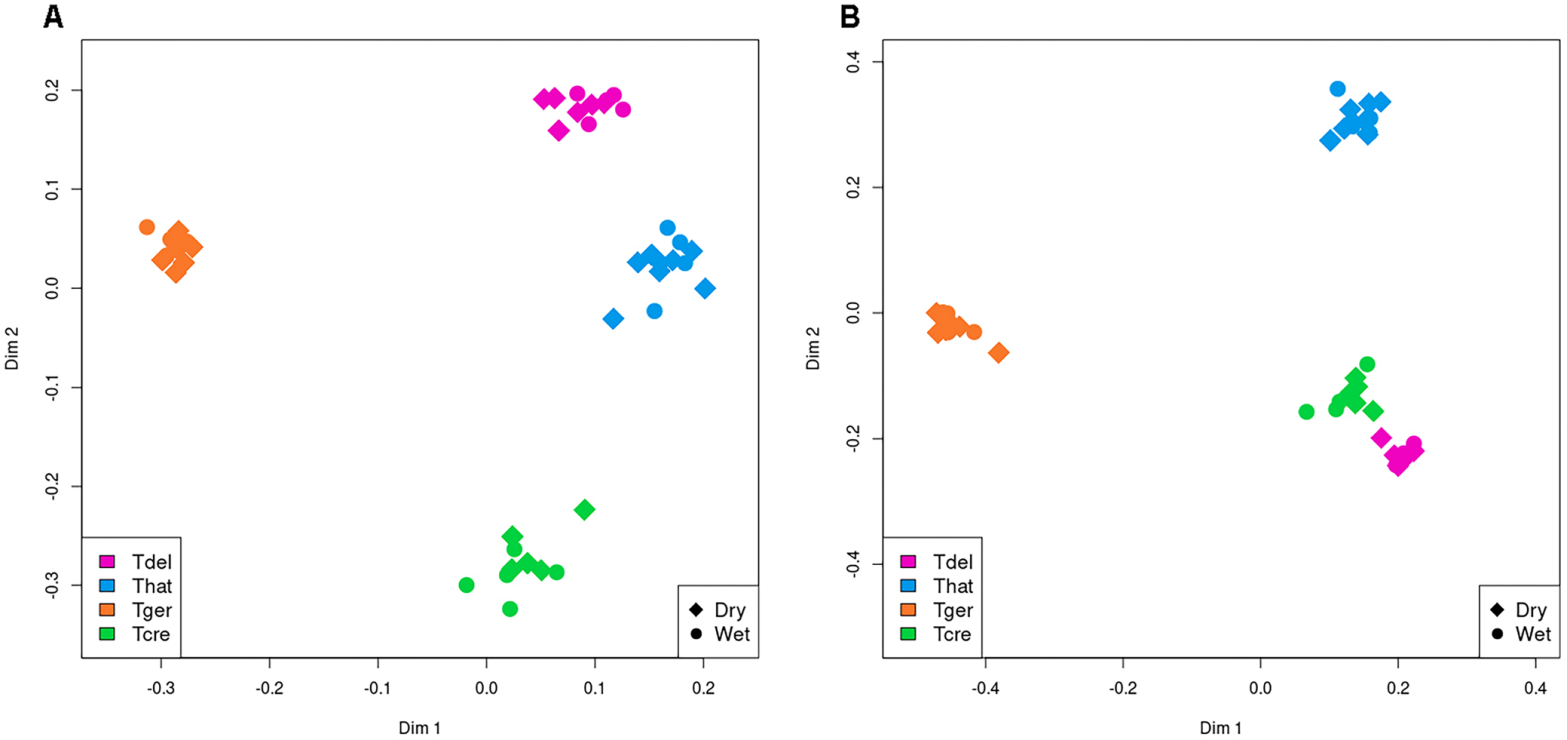
Multidimensional Scaling of the Spearman correlation‐based distances (D = (1‐rho)/2) for the monthly averaged values for each species based on (A) plant family and (B) plant genus data.

We used nonmetric multidimensional scaling (NMDS) based on Spearman distance to assess whether plant taxa were differentially distributed between the four langur species. This analysis showed differential distribution of different plant taxa across the samples on both family (Figure 2A) and genus level (Figure 2B), but clustering of monthly samples for each langur species. In the analysis at plant genus level, *T. delacouri* and *T. crepusculus* were found rather close together (Figure 2B), suggesting that the diets of these two species are relatively similar. However, this pattern was not observed on plant family level, which is probably due to the fact that around 50% of the diet of *T. crepusculus* was identified as belonging to the family Fagaceae, a family not found in *T. delacouri* . Since these approximately 50% of reads could not be identified down to genus level and are consequently not part of the genus level data set, the diet of these two langur species is found to be quite similar on genus level, but different on family level.

**FIGURE 3:**
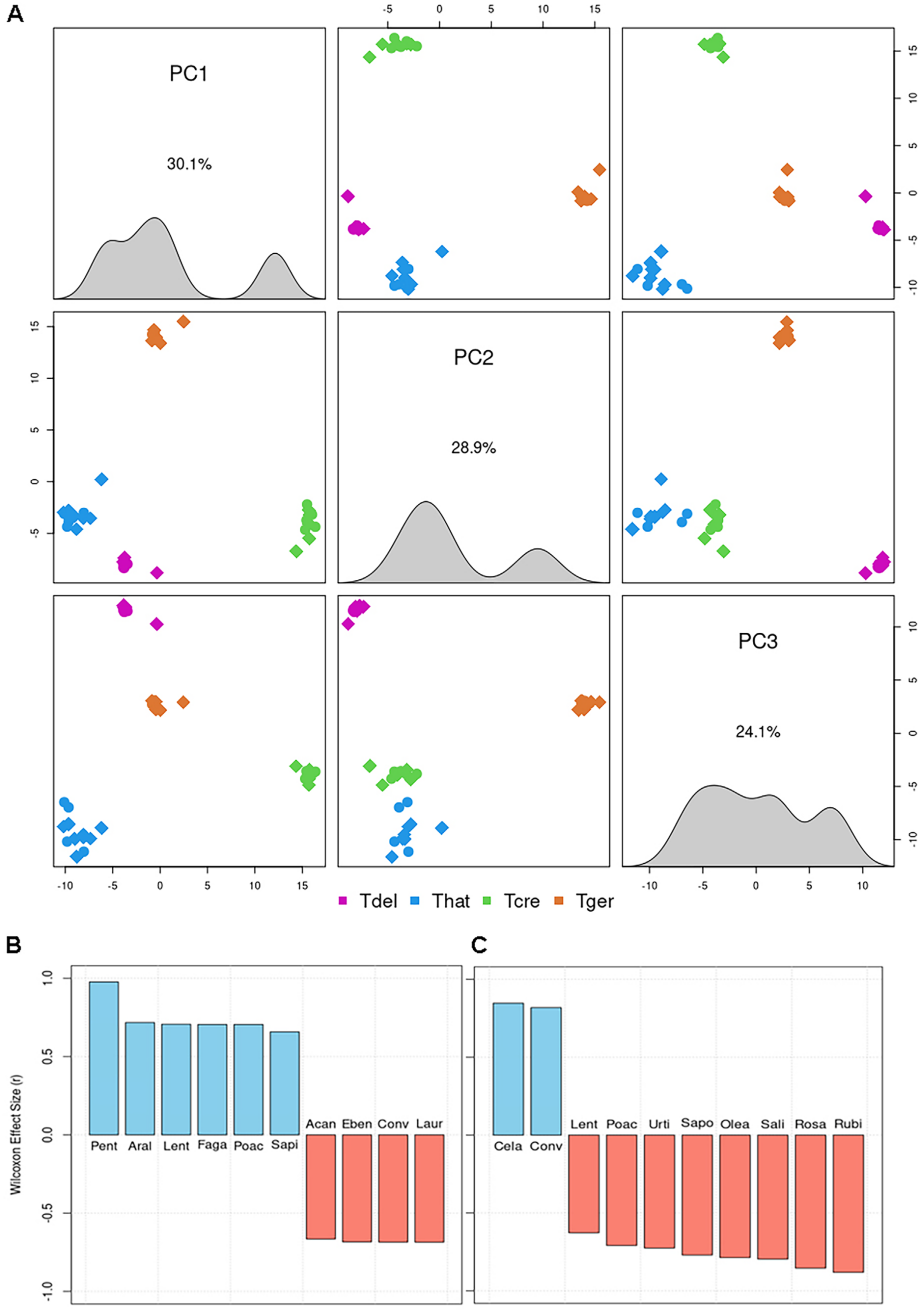
(A) PCA of plant family abundance data based on the log_2_(*x* + 1). Circles indicate data for wet season months, diamonds for dry season months. PC1 reflects the habitat component, distinguishing the rainforest species *T. crepusculus* from limestone‐habitat species. PC2 separates the rainforest langur living in limestone habitat ( *T. germaini* ) from the two limestone langurs. (B) Wilcoxon test effect size (*r*) for the comparison based on habitat ( *T. crepusculus* —rainforest habitat versus *T. delacouri* + *T. hatinhensis* + *T. germaini* —all limestone habitat). (C) Wilcoxon test effect size (*r*) for the comparison based on langur division ( *T. crepusculus* + *T. germaini* —both rainforest langurs versus *T. delacouri* + *T. hatinhensis* —both limestone langurs). For abbreviations of plant families, see Table S19.

A PCA on log_2_(*x* + 1) data revealed a similar picture of four distinct clusters at family and genus level. On family level, PC1 and PC2 together accounted for 59.0% of the total variation, while PC3 explained an additional 24.1% of the variance (Figure 3A, Figures S1 and S2). On genus level, PC1 and PC2 explained together 63.7% and PC3 another 20.2% (Figures S3 and S4). PC1 reflected the habitat component, distinguishing the rainforest species *T. crepusculus* from the limestone‐habitat species, whereas in PC2 the rainforest langur living in limestone habitat ( *T. germaini* ) is separated from the two limestone langurs. The Kruskal–Wallis test and post hoc pairwise Wilcoxon tests revealed significant differences among groups (*p* < 0.001), with large effect sizes observed across habitat types (ε^2^ ≥ 0.8).

Consistent with the MDS and PCA results, the Wilcoxon test Effect size (*r*) revealed clear compositional differences among the *Trachypithecus* groups (Table S2). Effect size analyses revealed distinct associations between habitat groups. Araliaceae, Fagaceae, Lentibulariaceae, Poaceae, Pentaphylacaceae, and Sapindaceae were associated with the rainforest living *T. crepusculus* , while Lauraceae, Convolvulaceae, Ebenaceae, and Acanthaceae were associated with the three langur species inhabiting limestone habitats (Figure 3B). In contrast, when considering the grouping of rainforest langurs versus limestone langurs, Celastraceae and Convolvulaceae were associated with the rainforest langurs ( *T. crepusculus* and *T. germaini* ), while Rubiaceae, Rosaceae, Salicaceae, Oleaceae, Sapotaceae, Urticaceae, Poaceae, and Lentibulariaceae were more strongly associated with the limestone langurs ( *T. delacouri* and *T. hatinhensis* ) (Figure 3C).

### 3.3 Variation in Dietary Diversity Among *Trachypithecus* Species

We further investigated plant diversity using family‐level assignments. Both Shannon diversity and species evenness indices demonstrated that alpha diversity of the diet was significantly different across the four langur species (Kruskal–Wallis: *X* ^2^ = 13.429, df = 3, *p* < 0.05; Table 2), although the effect size was small (ε^2^ = 0.035), indicating relatively modest interspecific differences. Additionally, beta diversity was also found to differ significantly among all four langur species (AMOVA: Bray–Curtis: *F* = 40.215, *p* < 0.001; Unweighted UniFrac: *F* = 40.566, *p* < 0.001), as well as between any specific pair of langurs (AMOVA: Bray‐Curtis: *p* < 0.001; Unweigted UniFrac: *p* < 0.001). The same is true for plant genus level data (Table 2).

**TABLE 2:** Summary of the alpha diversity indices at plant family and genus level, across all months.

Level	Indexes	Tdel	That	Tger	Tcre
Family	Observed	62	60	59	73
	Shannon	2.474	2.558	1.908	2.073
	Evenness	0.599	0.625	0.468	0.483
Genus	Observed	122	129	125	129
	Shannon	2.619	2.813	2.188	3.143
	Evenness	0.545	0.579	0.453	0.647

*Note:* Observed: The number of observed taxa/OTUs; Shannon: Shannon diversity index; Evenness: species evenness index; the latter two averaged across all months.

**FIGURE 4:**
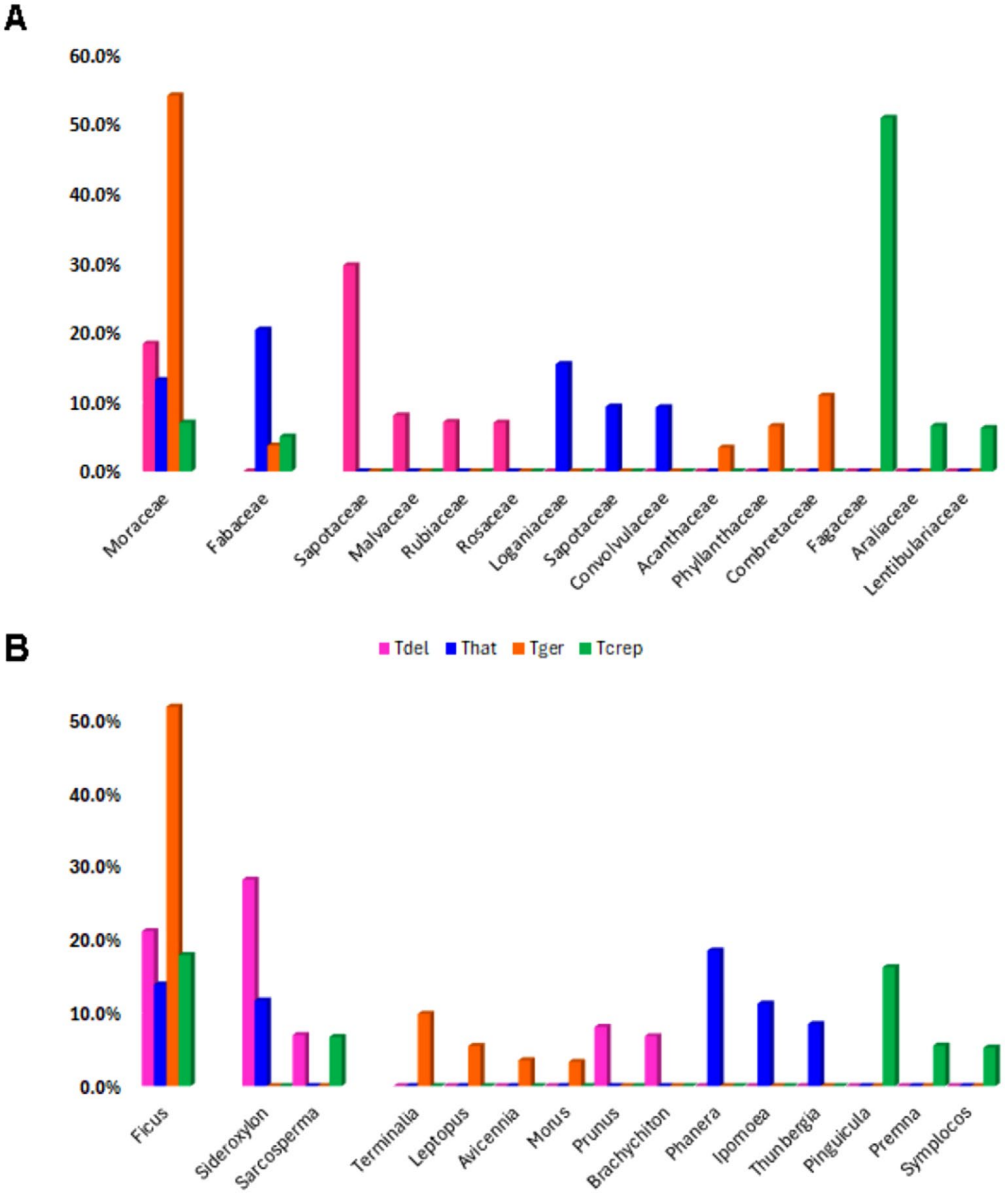
Relative abundance of the five most frequently consumed plant families (A) and plant genera (B) in the diet of each study species. On the family level, Moraceae was consumed by all four langur species and Fabaceae by three species, while all other most frequently consumed plant families were consumed only by a single langur species. On genus level, *Ficus* was consumed by all four langur species, and *Sideroxylon* and *Sarcosperma* by two species, while all other most frequently consumed plant genera were consumed only by a single langur species.

Overall, a wide range of plant families and genera contributed to the diet of the four langur species. When considering the five most consumed plant families for each langur species, Moraceae was consistently among the dominant dietary components across all langur species and constituted 7.0%–53.9% of langur diet (Figure 4A, Tables S3–, S6). Fabaceae was consumed by three langur species and contributed 3.7%–20.3% of the langur diet. The remaining plant families that were among the five most consumed ones were all found in this category for single langur species only, thereby contributing 3.4%–50.7% to the diet of the respective species (Figure 4A, Tables S3–, S6). The situation is similar on plant genus level. Among the five most consumed plant genera for each langur species, *Ficus* (family Moraceae) was found in all four langur species, contributing 13.8%–51.7% of their respective diets (Figure 4B, Tables S7–, S10). *Sideroxylon* was part of the diet of *T. hatinhensis* (11.6%) and *T. delacouri* (28.1%), and *Sarcosperma* was consumed by *T. delacouri* (6.9%) and *T. crepusculus* (6.7%). Again, as on plant family level, most of the five most commonly consumed plant genera are in this category only found in a single langur species, thereby contributing 3.3%–18.5% to the diet of the respective species (Figure 4B, Tables S7–, S10).

Over the year, the diet of *T. delacouri* consisted of 122 plant genera (45 genera with abundance ≥ 0.1%; Table S7), belonging to 62 families (33 with an abundance ≥ 0.1%; Table S3). The contribution of the five most common plant families varied from 7.0% to 29.6%, summing up to 70% of the total feeding record (Figure 4A, Table S3). The most dominant genera were *Sideroxylon* with a proportion of 28.1% and *Ficus* (21.1%), followed by *Prunus* (8.0%), *Sarcosperma* (6.9%), and *Brachychiton* (6.8%), making up 71.0% of the total feeding record of *T. delacouri* (Figure 4B, Table S7).

For *T. hatinhensis* we found that its diet contained a total of 129 plant genera of which only 41 had an abundance ≥ 0.1% (Table S8). The identified genera could be assigned to 60 families, roughly half of which (28 families) had an abundance ≥ 0.1% (Table S4). The five most consumed plant families varied from 9.2% to 20.3%, and summed up to a total of 67.3% (Figure 4A, Table S4). The five most common plant genera in the diet of *T. hatinhensis* were *Phanera* (16.4%), *Ficus* (13.1%), *Sideroxylon* (11.2%), *Ipomoea* (10.7%), and *Thunbergia* (8.1%), which make up 59.5% of this species' food composition (Figure 4B, Table S8).

For *T. germaini* we found 125 plant genera of which 36 had an abundance ≥ 0.1% (Table S9). These plant genera belong to 59 families (abundance of 27 families ≥ 0.1%, Table S5). The contribution of the five most common plant families varied from 3.4% to 53.9%, totaling 78.4% of the diet (Figure 4A, Table S5). While the most dominant genus in *T. germaini* was *Ficus* with an average proportion of 51.7%, the next four mostly consumed genera contributed much smaller amounts to its diet: *Terminalia* (9.9%), *Leptopus* (5.5%), *Avicennia* (3.5%), and *Morus* (3.3%), which together sum up to 73.8% of the total diet (Figure 4B, Table S9).

The diet of *T. crepusculus* consisted of 129 plant genera with 52 genera having an abundance ≥ 0.1% (Table S10). The identified genera were assigned to 73 families of which 41% (30 families) had an abundance ≥ 0.1% (Table S6). In this langur species, the five most commonly found plant families varied from 5.0% to 50.7%, accounting for 75.5% of the diet (Figure 4A, Table S6). The five most common genera in the diet of *T. crepusculus* were *Ficus* (17.9%), *Pinguicula* (16.2%), *Sarcosperma* (6.7%), *Premna* (5.5%), and *Symplocos* (5.2%), summing up to 51.5% of the total diet (Figure 4B, Table S10). It should be noted that *T. crepusculus* fed largely on Fagaceae (50.7%), but as we were unable to assign sequence reads to Fagaceae genera, only non‐Fagaceae plant genera appear among the five most consumed plant genera.

### 3.4 Seasonal Dietary Composition

**FIGURE 5:**
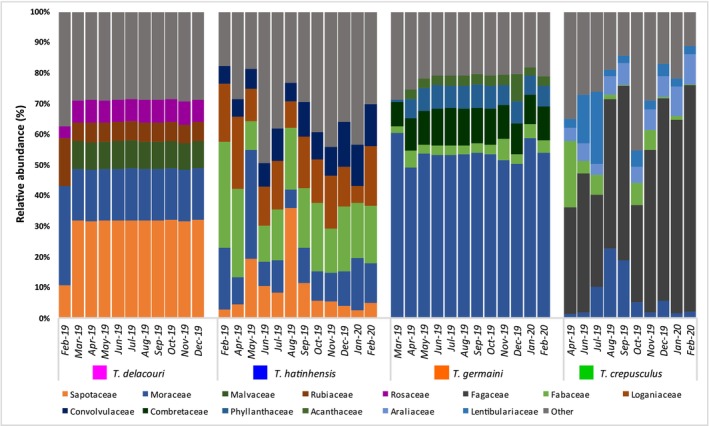
Relative abundance of the top five plant families in the diet of the four langur species over a period of 12 consecutive months (note: For *T. delacouri* and *T. crepusculus* , data are only available for 11 and 10 months, respectively). Stacked bar graphs describe the abundance of families, with the *x*‐axis representing the langur species in monthly time windows. For plant genera data see Figure S6.

When we compared the most abundant plant families consumed by langurs on a monthly and seasonal (wet and dry) basis, we found different degrees of variation. We found substantial monthly and seasonal variation in *T. hatinhensis* and *T. crepusculus* with the largest variation observed in *T. crepusculus* . In contrast, *T. delacouri* and *T. germaini* showed little differences in diet over the year (Figure 5, Figure S5, Tables S11–, S14). In *T. hatinhensis* , feeding on the most common family (Fabaceae) peaked at 34.7% in February but ranged from 9.2% to 28.9% in other months. Similar patterns were seen for Loganiaceae (5.6%–23.7%), Moraceae (6.0%–35.6%), Sapotaceae (2.5%–35.9%), and Convolvulaceae (5.7%–14.7%) (Table S11). In *T. crepusculus*, Fagaceae dominated the diet but fluctuated sharply between months (29.9%–73.9%), alongside variable contributions from Moraceae, Araliaceae, Lentibulariaceae, and Fabaceae (Table S12). In contrast, *T. delacouri* and *T. germaini* showed much less seasonal differences with almost no differences in consumed plant families in the wet and dry season (Figure 5, Figure S5, Tables S13 and S14). The diet of *T. delacouri* was consistently dominated by Sapotaceae and Moraceae, with smaller but steady inputs from Malvaceae, Rubiaceae, and Rosaceae; only for February‐19 we observed slight changes with less Sapotaceae and more Moraceae (Table S13). *Trachypitheus germaini* fed heavily and consistently on Moraceae (49.3%–60.6%), followed by Combretaceae (7.8%–12.3%) and minor contributions from other families (Table S14).

At the genus level, the seasonal patterns were generally similar to those recorded for the plant families, with some genera showing marked fluctuations between months and seasons, while others remained stable and dominant throughout the year (Figures S6 and S7, Tables S15–, S18). In *T. hatinhensis* , the proportion of the most consumed genus *Phanera* varied markedly throughout the year, reaching its highest contribution to the overall diet in February (42.3%) and April (31.4%), and ranging from 8.3% to 21.5% in other months (Figures S6 and S6, Table S15). In May, *Ficus* reached a similarly high proportion (39.5%) and remained within a comparable range (5.1%–23.3%) for the rest of the year. *Sideroxylon*, although less dominant overall, became the most consumed genus in August (40.9%), far exceeding its contribution in other months (2.8%–20.7%). *Ipomoea* showed moderate but consistent presence year‐round (6.6%–17.4%), with peaks in December (16.5%), January (14.1%), and February 2020 (17.4%). *Thunbergia* was generally a minor dietary component (4.0%–9.7%) except for a notable spike in January (29.7%).

In *T. crepusculus* , *Ficus* also exhibited strong seasonal variation, with very high proportions in August (51.6%) and September (52.9%), but much lower values in most other months (2.6%–21.5%). The genus *Pinguicula* also had a high proportion in the wet season, particularly in June (39.1%) and July (41.2%), while remaining below 18.1% in other months (Figures S6 and S7, Table S16). *Sarcosperma* was dominant in April (23.0%) and November (13.3%) but contributed much less (< 10.3%) during the rest of the year. *Premna* was a minor dietary component overall but increased notably in June (11.8%), July (13.1%), and August (14.8%). *Symplocos* appeared only sporadically, with high contributions in November (24.4%) and October (17.0%), while it was not recorded in most months.

The situation is different for *T. germaini* . Similar to the analysis on plant family level, we found little variation between wet and dry season in the proportion of the most common genus, which was again *Ficus*, for which the highest diet contribution was recorded in March (60.5%), while the lowest was in April, that is, in the immediately following month with still 47.1%, corresponding to a factor of just 1.4 between highest and lowest month (Figures S6 and S7, Table S17). Similarly, for *T. delacouri* , we also found limited variation between dry and wet season, as the highest proportion of the overall most common genus, *Sideroxylon* with a total abundance of 28.1%, showed abundances between 29.5% and 30.2% for all months except February 2019, when its abundance dropped to 11.7% (Figures S6 and S7, Table S18). In this month, the overall second most common genus (*Ficus*) in the diet of *T. delacouri* was recorded at 42.4%, while in all other months it was ranging at around 19.0% (Figures S6 and S7, Table S18).

Finally, when we tested alpha diversity as represented by Shannon diversity and species evenness indices, we found that the overall dietary diversity between dry and wet seasons was not significantly different across the four langur species (Kruskal–Wallis test, *p* > 0.05, ε^2^ = 0.009). Furthermore, the AMOVA results at the family level also indicated no significant difference in dietary diversity between dry and wet seasons (AMOVA: Bray–Curtis: *F* = 0.476, *p* = 0.812; Unweighted UniFrac: *F* = 0.877, *p* = 0.493). Similarly, at the genus level, there was no difference between dry and wet seasons (AMOVA: Bray–Curtis: *F* = 0.675, *p* = 0.685; Unweighted UniFrac: *F* = 0.773, *p* = 0.577). Thus, although at least some of the langur species consume different plants during dry and wet season, the overall dietary diversity remained unchanged.

## 4 Discussion

Revealing the diet of wild animal species is key to better understand their ecology. Previous genomic evidence from limestone langurs has demonstrated adaptive responses to specific environmental conditions, including high calcium intake (Liu et al. 2020; Liu et al. 2025; Zhang et al. 2024). Our results indicate variation in dietary composition among species and habitat‐associated groups, reflecting ecological associations rather than direct measures of food availability or habitat quality. In particular, langurs appear to exhibit considerable dietary flexibility in relation to locally available plant resources, as inferred from compositional dietary data. This is particularly evident by the herein studied rainforest langur *T. germaini* population which lives in limestone habitat and which is more similar in food plant composition to limestone langurs than to the rainforest langur living in a rainforest habitat, *T. crepusculus* .

Revealing the diet of wild animal species is not trivial, since observing what they feed on is often challenging, time consuming, and imprecise. Our study successfully applied advances in DNA metabarcoding to achieve a greater understanding of the dietary diversity in four *Trachypithecus* species occupying different habitats (limestone vs. rainforest habitats). This approach can be widely used for several applications related to conservation and ecology in the future. However, our results also show that identifications on different taxonomic levels can lead to substantially different conclusions. For instance, on the family level, we found that around half of the diet of *T. crepusculus* consists of the family Fagaceae, but this family is not represented on the genus level, as the corresponding reads could not be identified to genus level. Due to these differences in identification, the same primary data set shows a highly similar diet for *T. crepusculus* and *T. delacouri* on the genus level, whereas the two langur species differ substantially on the family level. This result highlights how analyses on different taxonomic levels can result in substantially different outcomes, and conclusions should be drawn with caution. Additionally, an important limitation of dietary metabarcoding is the incompleteness of reference databases, which can constrain taxonomic resolution and partly explain variation in taxon assignment (Keck et al. 2023). Finally, we recognize that the absence of vegetation surveys and phenological data limits our ability to directly evaluate habitat‐driven mechanisms underlying dietary variation. Future studies incorporating quantitative vegetation assessments and phenological monitoring, together with dietary metabarcoding and integrative analytical approaches, will be necessary to disentangle the relative roles of species identity, habitat type, and site‐level environmental variation.

Our findings confirm a number of plant taxa reported in previous studies employing traditional observation methods as part of the diet of the four *Trachypithecus* species. At the same time, we expand the list of food plants known to be consumed by the langur species at both family and genus levels. For example, while a previous study (Workman 2010) detected 21 families and 36 genera as food plants of *T. delacouri* in Van Long Nature Reserve, we found at the same site 48 families and 98 genera (abundance ≥ 0.01%, Tables S7 and S21). Since the taxa overlap only partially between the two studies (14 families and 11 genera), this increases the total number of plant taxa detected as potential food plants of *T. delacouri* from 21 to 55 families and from 36 to 123 genera. For *T. hatinhensis* , we detected 98 plant genera from 46 families with abundance ≥ 0.01%, while a previous study (Truong 2013) in Phong Nha‐Ke Bang National Park revealed 22 families and 32 genera (Table 3, Tables S8 and S22). Fifteen plant families, but only four plant genera, overlap among these two studies, increasing the total number of potential food plants of *T. hatinhensis* to 53 families and 126 genera. Hai et al. (2017) studied food plants in *T. crepusculus* and reported 17 plant genera from 12 families in Xuan Lien National Park for this langur species. In contrast, we detected in our analysis, conducted at the same site, 111 plant genera from 59 families, of which only four genera overlap (Table 3, Tables S6 and S23). Based on Hai et al. (2017) and our study, *T. crepusculus* feeds in Xuan Lien National Park on at least 124 plant genera and 64 families. For *T. germaini* , we found in our study conducted at Chua Hang 91 plant genera from 46 families (Table 3, Tables S9 and S24). Le et al. (2019) performed an observational study at the same site and revealed 47 genera from 32 families. Using samples from Chua Hang, Ang (2016) applied a metabarcoding approach targeting the P6 loop of the plant chloroplast *trnL* intron and found 19 plant genera and a total of 20 families. Overall, the overlap of identified plant families and genera among the studies is limited with only 12 and seven identical genera identified in our study and Le et al. (2019) and Ang (2016), respectively, five genera between the latter two studies, and only three genera found in all three studies. At another site, Khoe La, which is, as Chua Hang, part of the Kieng Luong Karst Area, but more degraded because of limestone quarrying (Ang 2016), Duc et al. (2010) observed *T. germaini* feeding on 22 plant genera from 18 families and Ang (2016) detected 13 plant genera and a total of 16 families using metabarcoding; three plant genera were identical in both studies. Overall, the comparison among studies and study sites suggests that langur species may exhibit dietary flexibility in response to variation in food plant availability.

**TABLE 3:** Number of food plants on family and genus level consumed by the four *Trachypithecus* species as revealed by this study (abundance ≥ 0.01%) in comparison to earlier studies.

Species	Family	Genus	Method	Source
*T. delacouri* (Van Long)	48	98	Molecular	This study
*T. delacouri* (Van Long)	21	36	Observation	Workman (2010)
*T. hatinhensis* (Tuyen Hoa)	46	98	Molecular	This study
*T. hatinhensis* (Phong Nha‐Ke Bang)	38	No data	Observation	Haiha et al. (2013)
*T. hatinhensis* (Phong Nha‐Ke Bang)	22	32	Observation	Truong (2013)
*T. germaini* (Chua Hang)	46	91	Molecular	This study
*T. germaini* (Khoe La)	18	22	Observation	Duc et al. (2010)
*T. germaini* (Chua Hang)	20	19	Molecular	Ang (2016)
*T. germaini* (Khoe La)	16	13	Molecular	Ang (2016)
*T. germaini* (Chua Hang)	32	47	Observation	Le et al. (2019)
*T. crepusculus* (Xuan Lien)	59	111	Molecular	This study
*T. crepusculus* (Xuan Lien)	12	17	Observation	Hai et al. (2017)

The semiquantitative approach applied in our study nevertheless allows some conclusions regarding the most important food plant groups, at least regarding the langur populations investigated in this study. Thus, the most common plant genus in our data set was the genus *Ficus* (Moraceae) that plays a key role in the diet of all four langur species. According to our data, the respective family Moraceae also plays a key role on the family level, but this may largely be driven by the langurs feeding on *Ficus* plants (Lim et al. 2021). With currently 884 accepted species worldwide (https://powo.science.kew.org/taxon/urn:lsid:ipni.org:names:327905‐2#children), *Ficus* is not only the genus with the largest number of species within Moraceae (Shanahan et al. 2001; Clink et al. 2017), but also among flowering plants in general (Judd et al. 2007). *Ficus* is an important component of tropical forest ecosystems and to a lesser extent, subtropical regions and its members are recognized as keystone species because they provide an essential food resource for frugivorous animals (Berg and Corner 2005; Dev et al. 2011). Our data suggests that this key role extends beyond frugivorous species also to leaf‐eating ones. In particular, our results revealed that *Ficus* made up more than 50% of the food composition of *T. germaini* , which is in agreement with earlier studies that *Ficus* constitutes a significant dietary component of this langur species (Ang 2016; Le et al. 2019).

Comparative studies of folivorous primates show that dietary strategies are largely shaped by environmental variation and resource availability. Limestone landscapes are widely recognized as ecosystems of high biological importance due to their high levels of endemism and unique flora and fauna (Fernando et al. 2008; Struebig et al. 2009), with vegetation ranging from stunted trees on exposed cliffs to taller forest on gentler slopes, reflecting strong spatial heterogeneity in local environmental conditions (Fernando et al. 2008). Our results are consistent with previous findings indicating that *Trachypithecus* species exploit a broad range of plant taxa within their habitats, although reliance on key resources varies with ecological context. In limestone forests, vegetation is often patchily distributed, which may limit food resource diversity while maintaining relatively predictable access to certain key plant species. Accordingly, Francois' langur ( *T. francoisi* ) populations show high folivory and strong reliance on a limited subset of plant species despite overall dietary diversity, reflecting the constraints of relatively stable but resource‐limited limestone environments, where food availability is predictable but plant diversity is relatively restricted (Zhou et al. 2006; Huang et al. 2008; Zhou et al. 2018; Yao et al. 2024). In contrast, snub‐nosed monkeys (*Rhinopithecus* spp.) inhabiting temperate forests exhibit pronounced seasonal dietary shifts driven by temporal variation in resource availability, where higher plant diversity is associated with strong seasonality in food production (Grueter et al. 2009; Hou et al. 2018). Similar patterns are also observed among African colobines, highlighting how habitat structure influences dietary flexibility and reliance on key resources. For example, *Colobus guereza* shows a highly folivorous diet with strong dependence on leaves, consistent with relatively stable forest conditions that may promote dietary specialization. By contrast, *Piliocolobus badius* exhibits greater dietary breadth and flexible resource use, particularly in heterogeneous rainforest environments where food resources are diverse but spatially and temporally variable (Clutton‐Brock 1975; Harris and Chapman 2007). Consistently, Aleixo‐Pais et al. (2023), using a metabarcoding approach based on noninvasive samples, identified 97 plant taxa in the diet of *P. badius* (62 taxa in the Gola Rainforest National Park, Sierra Leone population and 45 taxa in the Cantanhez National Park, Guinea‐Bissau), although only four to five taxa were most frequently detected in each population, indicating a concentration on a small number of key resources despite broad dietary sampling.

The two plant families Moraceae and Fabaceae are consumed by almost 75% of primate species (Lim et al. 2021). Therefore, the key role of Moraceae, which we found to be among the three most commonly consumed plant families in all four langur species (Tables S3–, S6) is not surprising. In contrast, Fabaceae plays a less important role in the diet of the four langur species with contributions of 1.2% to 18.1% to the langurs' diet (Table S3–, S6). No other plant family was among the five most common ones in all our langur species and only the family Sapotaceae was also detected in all four langur species, albeit with variable contributions of 0.2% to 29.6% to the langurs' diet. However, it was generally detected in smaller numbers than Fabaceae, except for *T. delacouri* , where it is the main diet item on the family level (29.6%).

Thus, beyond the key role of *Ficus*, we found that overall, all four langur species display substantial differences in their diet (Tables S3–, S10). However, since detailed quantitative data on the abundance of the different plant species in the respective habitats are not available, we cannot decide whether these differences in diet are due to different dietary preferences, environmental variables such as food availability in their habitats or a combination of these two factors. Therefore, differences in plant composition and overall dietary diversity among langur species may be influenced by dietary preferences, habitat variation, or a combination of both factors. Shannon diversity index and evenness index differ significantly among the four species; however, effect sizes were generally small (e.g., ε^2^ = 0.035), suggesting that interspecific variation in dietary diversity was modest.

We also found differences between the species regarding monthly and seasonal variation. *Trachypithecus hatinhensis* and *T. crepusculus* showed some variation, whereas we observed little seasonal variation in the plants consumed by *T. delacouri* and *T. germaini* (Tables S11 and S12). This suggests that the dietary habits of *T. hatinhensis* and *T. crepusculus* are influenced by the availability of certain plant species during different seasons. Whether it is the availability of plants alone or rather a combination of plant availability and dietary flexibility can, in the absence of vegetation data from all habitats, currently not be decided. In contrast, *T. delacouri* and *T. germaini* have a more consistent diet, regardless of the season. However, it remains unclear whether this pattern reflects more seasonal habitat conditions or whether these langurs maintain relatively consistent diets independent of the quantitative availability of plant resources. Thus, the observed differences in dietary patterns among langur species may be influenced by multiple factors, including habitat characteristics and ecological niche differentiation (Schoener 1971; Marshall et al. 2009), as well as potential responses to environmental variation (Chapman et al. 2013). As a next step, quantitative vegetation data would be valuable to further evaluate these alternative explanations.

While our findings suggest that the investigated langur species consume a wide range of food plant resources, the genus *Ficus* consistently played a key role in the diet of all species, a conclusion further supported by the fact that it was also discovered in all except one of the previous studies on langur diet (Lai et al. 2024) (Tables S7–, S10). This finding suggests that species of the genus *Ficus* constitute an important food resource for these langurs across their range, regardless of their geographical location or habitat. Vietnam spans six distinct bioclimatic regions, characterized by substantial variation in climate, vegetation structure, and floristic composition. Despite this environmental heterogeneity, *Ficus* was consistently detected in the diet, underscoring its role as a broadly available and reliable food source for langurs. However, it is important to note that plant identification in this study was generally limited to the genus level, which precludes conclusions about the consumption of individual *Ficus* species. This limitation is particularly relevant given that not all *Ficus* species are equally consumed by a given langur species, dietary preferences may vary according to local plant availability, ecological conditions, and dietary selectivity within the genus *Ficus* has been widely documented in primates (Serio‐Silva et al. 2002; Clink et al. 2017). On the other hand, our data also revealed that even consumption of *Ficus* as the most common food item overall, varies seasonally. Again, vegetation data are required to find out whether this variation is due to the seasonally variable availability of certain plant parts for consumption like young leaves or shoots or has other causes like replacement of *Ficus* by other, only seasonally available plant species. Differentiating between these two possibilities would be important, as the former use would identify *Ficus* as a preferred food item, whereas the latter would identify it as a “fallback‐option” in cases when other and preferred food items are not available. Consequently, while our results highlight the importance of *Ficus* at the genus level, future studies combining molecular dietary analysis with detailed botanical surveys are needed to resolve species‐level dietary specialization across different bioclimatic regions.

Beyond comparisons among species, differences between limestone and rainforest habitats may also help explain variation in dietary patterns among langurs. Limestone habitats are often characterized by relatively low plant diversity but can provide stable and predictable food resources throughout the year, which may encourage greater reliance on a limited number of key food plants (Huang et al. 2008; Zhou et al. 2018; Yao et al. 2024). At the same time, the restricted diversity and patchy distribution of vegetation in karst landscapes may limit feeding options and increase dependence on particular plant taxa. In contrast, rainforest habitats generally support a greater diversity of plant species and therefore offer a wider range of potential food resources (Richards 1996). This may allow langurs to exploit a broader range of food plants and adjust their diets more flexibly according to local conditions. However, food availability in rainforests can also vary seasonally and spatially, requiring animals to modify their feeding behavior in response to changing resource patterns (Grueter et al. 2009; Hou et al. 2018). Although our results did not show a clear separation in dietary composition between limestone and rainforest species, these habitat‐related differences may contribute to the variation in food plant diversity and resource use reported across *Trachypithecus* populations. Future studies combining dietary data with vegetation surveys and phenological monitoring would help clarify how habitat characteristics influence feeding ecology and dietary adaptation in these folivorous primates.

Overall, the findings of our study suggest that the dietary strategies of the studied *Trachypithecus* species are relatively flexible, consistent with patterns reported in other folivorous colobines as they have the ability to exploit a broad range of plant taxa and adjust their feeding behavior across ecological contexts. Moreover, our results do not provide evidence for a strong separation in dietary composition between habitat types as shown for the investigated rainforest langur *T. germaini* living in a limestone habitat. However, our study is subject to several limitations, including the relatively small number of species examined and partially constrained comparisons across habitat types due to the study design. While metabarcoding provides detailed taxonomic resolution of diet composition, more comprehensive ecological interpretation would benefit from integration of vegetation composition and food availability data. In addition, variation in sampling design, seasonal coverage, and methodological approaches across studies may limit direct comparability of dietary data. Future research should therefore integrate long‐term ecological and phenological monitoring, quantitative vegetation assessments, and nutritional or metabolomic approaches to better resolve the mechanisms underlying dietary variation and ecological adaptation. Despite these limitations, this study provides one of the most comprehensive datasets on food plant diversity for four *Trachypithecus* species. The results offer baseline information relevant for conservation planning, particularly in identifying commonly consumed plant taxa that may inform habitat management and restoration strategies in fragmented landscapes.

## Author Contributions

**L. T. Anh:** data curation (supporting), writing – review and editing (equal). **X. Wang:** data curation (supporting), writing – review and editing (equal). **M. Li:** conceptualization (equal), funding acquisition (lead), methodology (equal), supervision (lead), writing – original draft (equal). **M. Hofreiter:** conceptualization (equal), funding acquisition (lead), methodology (equal), project administration (equal), supervision (lead), writing – original draft (equal), writing – review and editing (equal). **C. Roos:** conceptualization (equal), funding acquisition (lead), methodology (equal), supervision (lead), writing – original draft (equal). **L. Hallmaier‐Wacker:** data curation (supporting), writing – review and editing (equal). **D. Groth:** data curation (equal), formal analysis (equal), methodology (equal), writing – review and editing (equal). **L. Zhang:** data curation (supporting), writing – review and editing (equal). **S. Knauf:** data curation (supporting), writing – review and editing (equal). **T. Nadler:** conceptualization (supporting), data curation (supporting), resources (supporting), writing – review and editing (equal). **L. Duc Minh:** conceptualization (supporting), data curation (supporting), writing – review and editing (equal). **A. Poehlein:** data curation (supporting), methodology (supporting), writing – review and editing (equal). **N. Van Truong:** conceptualization (equal), data curation (lead), formal analysis (equal), investigation (lead), methodology (equal), project administration (lead), resources (lead), writing – original draft (lead), writing – review and editing (equal).

## Funding

This research was provided by grants from the German Research Foundation to M.H. (HO 3492/9‐1) | C.R. (RO 3055/7‐1) | Chinese Academy of Sciences (CAS, XDB31000000) and National Natural Science Foundation of China (NSFC, 31821001) to M.L., and the Sino‐German Mobility Programme (M‐0084) to M.L. and C.R.

## Conflicts of Interest

The authors declare no conflicts of interest.

## Supporting information


**Figure S1:** PCA of plant family abundance data.
**Figure S2:** More detailed PCA (PC1‐5) of plant family abundance data based on the log_2_‐1 transformed *z*‐score data.
**Figure S3:** PCA of plant genus abundance data.
**Figure S4:** More detailed PCA (PC1‐5) of plant genus abundance data based on the log_2_‐1 transformed *z*‐score data.
**Figure S5:** Relative abundance of the top five plant genera among the four langur species studied over a period of 12 consecutive months.
**Figure S6:** Multidimensional Scaling of the Spearman correlation‐based distances (D = (1‐rho)/2) of plant genera for each langur species.

**Table S1:** Information about raw and filtered sequence data.

**Table S2:** Analysis of species pairs using molecular variance (AMOVA) at plant family and genus level.

**Table S3:** Diet diversity of *T. delacouri* at the plant family level.

**Table S4:** Diet diversity of *T. hatinhensis* at the plant family level.

**Table S5:** Diet diversity of *T. germaini* at the plant family level.

**Table S6:** Diet diversity of *T. crepusculus* at the plant family level.

**Table S7:** Diet diversity of *T. delacouri* at the plant genus level.

**Table S8:** Diet diversity of *T. hatinhensis* at the plant genus level.

**Table S9:** Diet diversity of *T. germaini* at the plant genus level.

**Table S10:** Diet diversity of *T. crepusculus* at the plant genus level.

**Table S11:** Diet diversity of *T. hatinhensis* at the plant family level by month.

**Table S12:** Diet diversity of *T. crepusculus* at the plant family level by month.

**Table S13:** Diet diversity of *T. delacouri* at the plant family level by month.

**Table S14:** Diet diversity of *T. germaini* at the plant family level by month.

**Table S15:** Diet diversity of *T. hatinhensis* at the plant genus level by month.

**Table S16:** Diet diversity of *T. crepusculus* at the plant genus level by month.

**Table S17:** Diet diversity of *T. germaini* at the plant genus level by month.

**Table S18:** Diet diversity of *T. delacouri* at the plant genus level by month.

**Table S19:** Abbreviations of plant families.

**Table S20:** Abbreviations of plant genera.

**Table S21:** Comparison of food plants (genus level) detected in our and an earlier study for *T. delacouri*.

**Table S22:** Comparison of food plants (genus level) detected in our and an earlier study for *T. hatinhensis* .

**Table S23:** Comparison of food plants (genus level) detected in our and earlier studies for *T. germaini*.

**Table S24:** Comparison of food plants (genus level) detected in our and an earlier study for *T. crepusculus* .

## Data Availability

The raw sequence data generated in this study were uploaded on NCBI and are available under bio project number PRJNA1051546 and the code is available from the R *trachy* package vignette (https://github.com/mittelmark/trachy).
