# Supplementary material for: Molecular Genetic Characterization of the Diet of Limestone and Rainforest Langurs
Source: Ecol Evol. 2026 Jun 30;16(7):e73892. doi: 10.1002/ece3.73892 (PMC13316972; doi:10.1002/ece3.73892)
Supplement: Supplementary file 1 — Figure S1: PCA of plant family abundance data. Figure S2: More detailed PCA (PC1‐5) of plant family abundance data based on the log2‐1 transformed z‐score data. Figure S3: PCA of plant genus abundance data. Figure S4: More detailed PCA (PC1‐5) of plant genus abundance data based on the log2‐1 transformed z‐score data. Figure S5: Relative abundance of the top five plant genera among the four langur species studied over a period of 12 consecutive months. Figure S6: Multidimensional Scaling of the Spearman correlation‐based distances (D = (1‐rho)/2) of plant genera for each langur species. [file ECE3-16-e73892-s023.docx]

**Supplementary information**

**Molecular genetic characterization of the diet of limestone and rainforest langurs**

N. V. Truong^1,2,3[
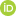
](https://orcid.org/0000-0003-2004-6658)^ | D. Groth^4[
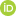
](https://orcid.org/0000-0002-9441-3978)^  | L. Hallmaier-Wacker^2[
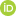
](https://orcid.org/0000-0002-0673-2275)^ | S. Knauf^5,6^[
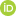
](https://orcid.org/0000-0001-5744-4946) | A. Poehlein^7[
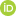
](https://orcid.org/0000-0002-2473-6202)^ | L. D. Minh^3[
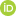
](https://orcid.org/0000-0002-2953-2815)^ | T. Nadler^8^ | L. Zhang^2,9[
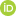
](https://orcid.org/0000-0002-8829-0588)^ | X. Wang^9^ | L. T. Anh^10^ | M. Li^9,11[
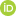
](https://orcid.org/0000-0001-5689-6270)^ | C. Roos^2,12[
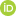
](https://orcid.org/0000-0003-0190-4266)^  | M. Hofreiter^1[
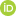
](https://orcid.org/0000-0003-0441-4705)^

^1^Evolutionary Adaptive Genomics, Institute of Biochemistry and Biology, Faculty of Science, University of Potsdam, Karl-Liebknecht-Str. 24-25, 14476 Potsdam, Germany | ^2^Primate Genetics Laboratory, German Primate Center, Leibniz Institute of Primate Research, Kellnerweg 4, 37077 Göttingen, Germany | ^3^Central Institute for Natural Resources and Environmental Studies, Vietnam National University, 19 Le Thanh Tong Street, Hanoi, Vietnam | ^4^Bioinformatics, Institute of Biochemistry and Biology, Department of Science, University of Potsdam, Karl-Liebknecht-Str. 24-25, 14476 Potsdam, Germany | ^5^Institute of International Animal Health/One Health, Friedrich-Loeffler-Institute, Federal Research Institute for Animal Health, Südufer 10, 17493 Greifswald - Insel Riems, Germany | ^6^Faculty of Veterinary Medicine, Justus Liebig University, Frankfurterstrasse 106, 35393 Giessen, Germany | ^7^Georg-August University of Göttingen, Institute of Microbiology and Genetics, Department of Genomic and Applied Microbiology, Grisebachstr. 8, 37077 Göttingen, Germany | ^8^Cuc Phuong Commune, Ninh Binh Province, Vietnam | ^9^CAS Key Laboratory of Animal Ecology and Conservation Biology, Institute of Zoology, Chinese Academy of Sciences, Beijing 100101, China | ^10^Mien Trung Institute for Scientific Research, Vietnam National Museum of Nature, Hue, Vietnam | ^11^Center for Excellence in Animal Evolution and Genetics, Chinese Academy of Sciences, Kunming 650223, China | ^12^Gene Bank of Primates, German Primate Center, Leibniz Institute for Primate Research, Kellnerweg 4, 37077 Göttingen, Germany

**Correspondences:** N. V. Truong ([truovannguyen@uni-potsdam.de](mailto:truovannguyen@uni-potsdam.de)) | C. Roos ([croos@dpz.eu](mailto:croos@dpz.eu)) | M. Hofreiter ([michael.hofreiter@uni-potsdam.de](mailto:michael.hofreiter@uni-potsdam.de))

**Funding: T**his research was provided by grants from the German Research Foundation to M.H. (HO 3492/9-1) | C.R. (RO 3055/7-1) | Chinese Academy of Sciences (CAS, XDB31000000) and National Natural Science Foundation of China (NSFC, 31821001) to M.L., and the Sino-German Mobility Programme (M-0084) to M.L. and C.R.

**Contents: Supplementary Figures S1 – S7**


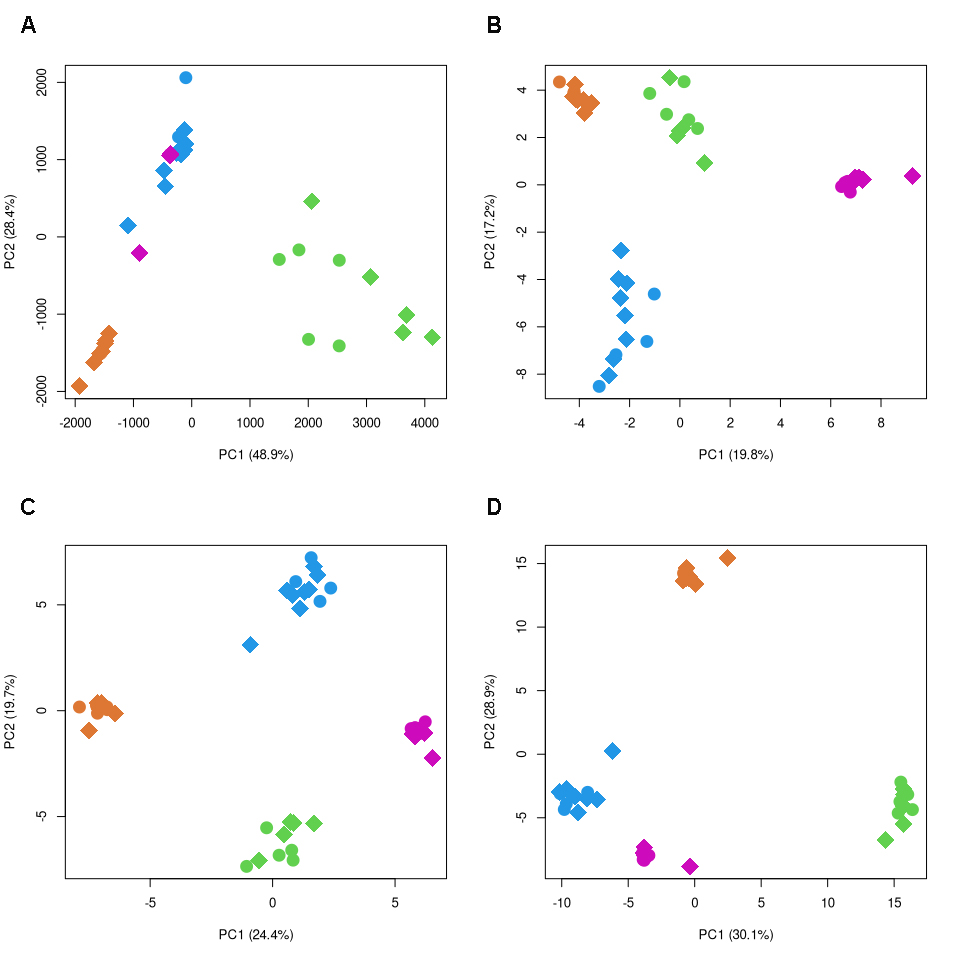


Figure S1. PCA of plant family abundance data. Shown are the results for **(A)** the raw data, **(B)** z-score scaled data, **(C)** log2+1 transformed z-score data and **(D)** log2-1 transformed z-score data. The most reliable approach, PCA based on log2-1 transformed z-score data, which retains the increased impact of high abundance plant families on the analysis, although damped due to the log transformation, shows a clear separation of langur species. Circles indicate data for wet season months and diamonds for dry season months.


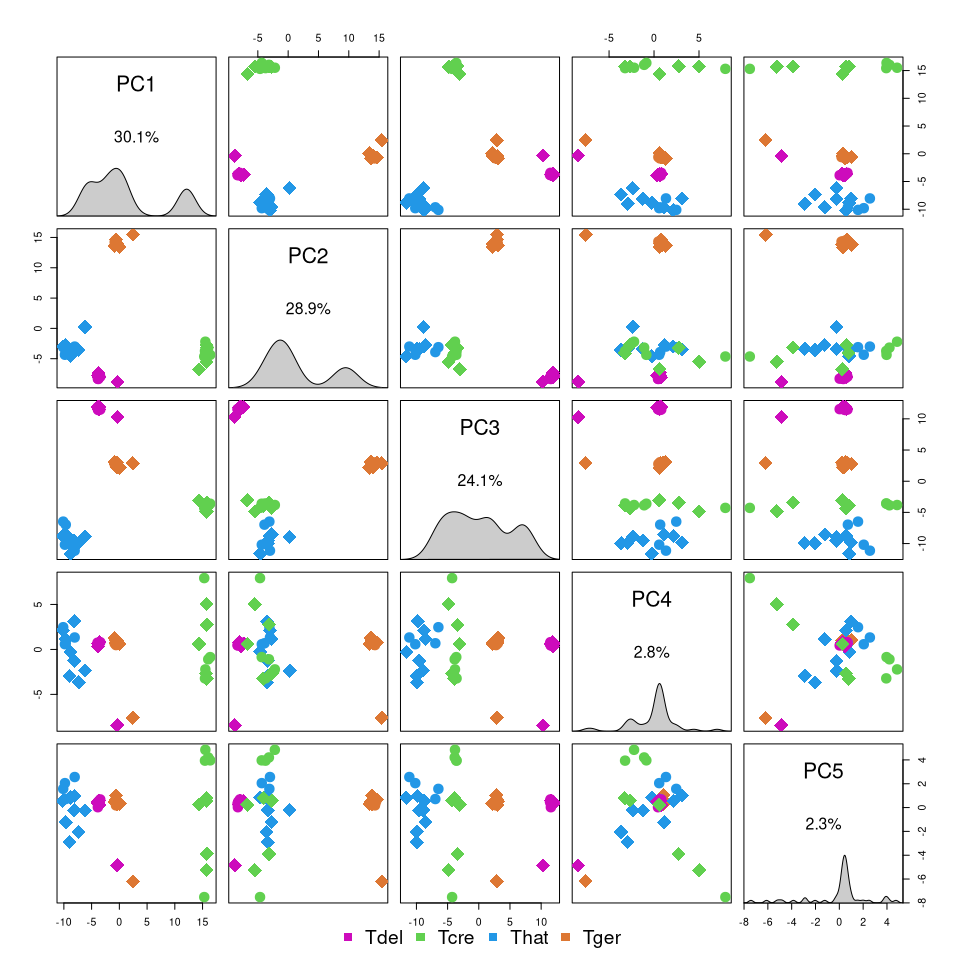


Figure S2. More detailed PCA (PC1-5) of plant family abundance data based on the log2-1 transformed z-score data. Circles indicate data for wet season months and diamonds for dry season months.


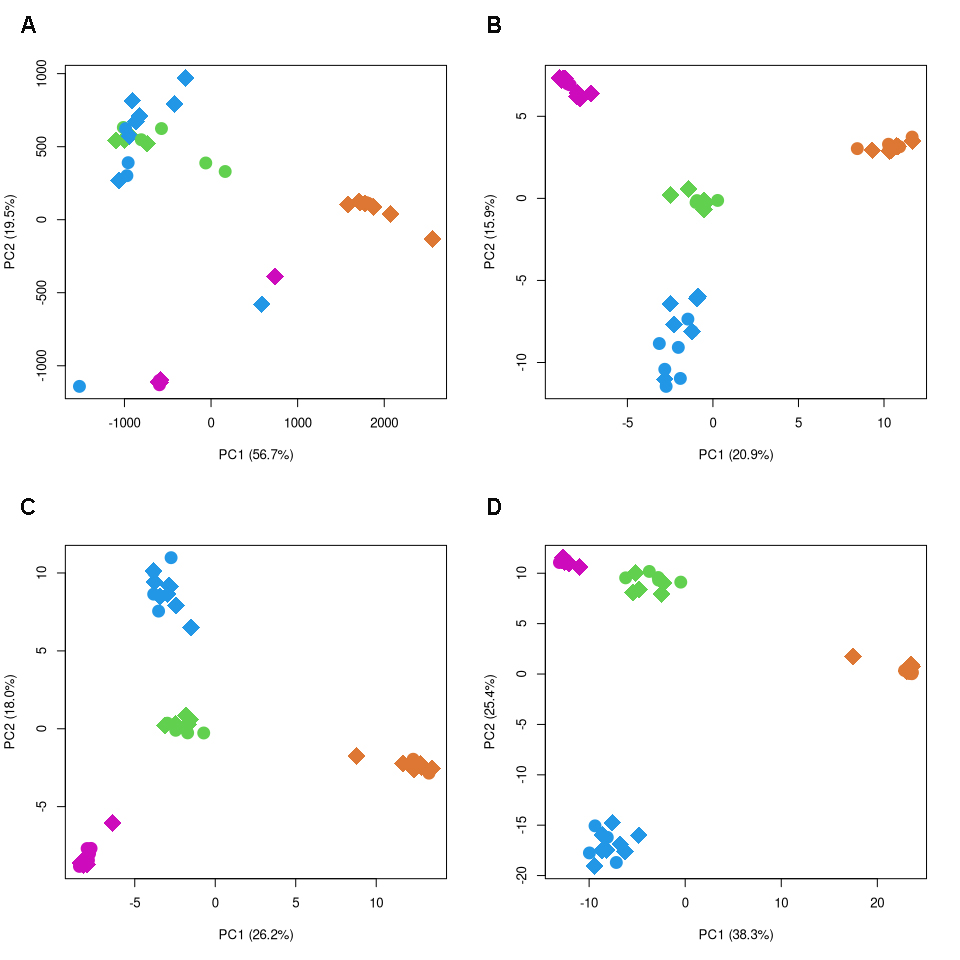


Figure S3. PCA of plant genus abundance data. Shown are the results for **(A)** the raw data, **(B)** z-score scaled data, **(C)** log2+1 transformed z-score data and **(D)** log2-1 transformed z-score data. The most reliable approach, PCA based on log2-1 on the abundance data, which retains the increased impact of high abundance plant genera on the analysis, although damped due to the log transformation, shows a clear separation of langur species. Circles indicate data for wet season months and diamonds for dry season months.


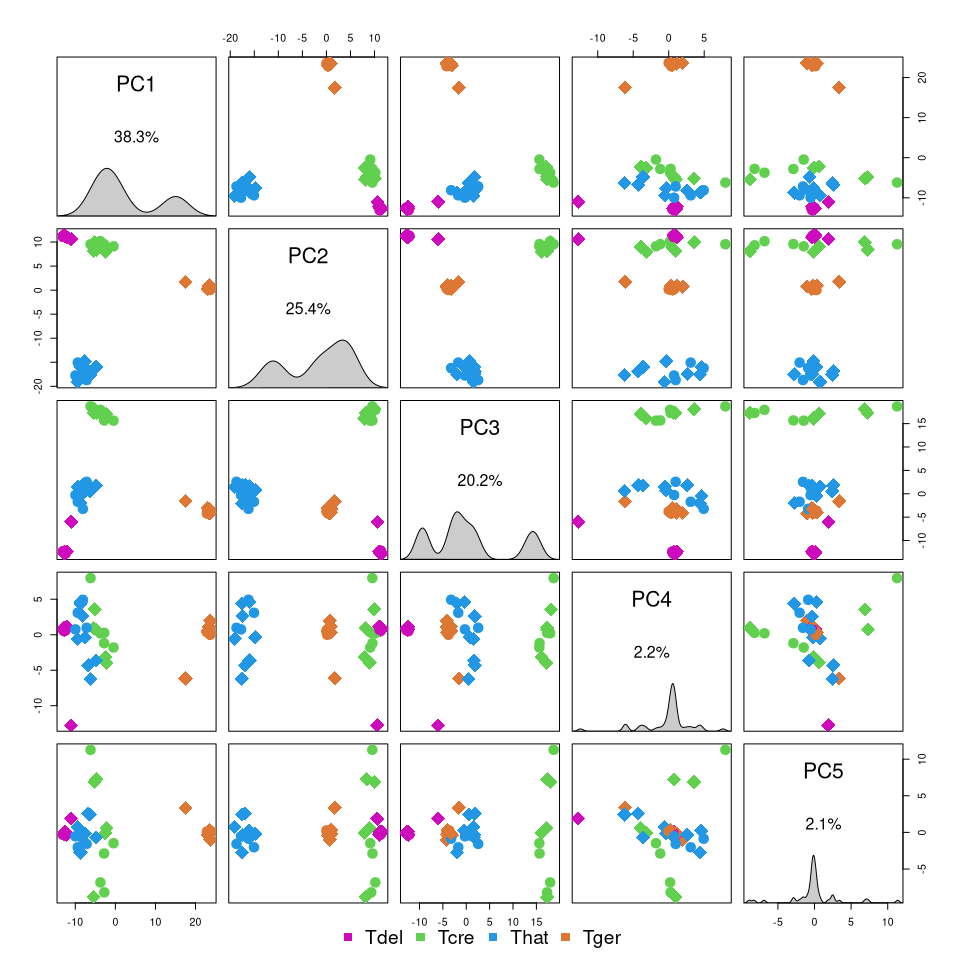


Figure S4. More detailed PCA (PC1-5) of plant genus abundance data based on the log2-1 transformed z-score data. Circles indicate data for wet season months and diamonds for dry season months.


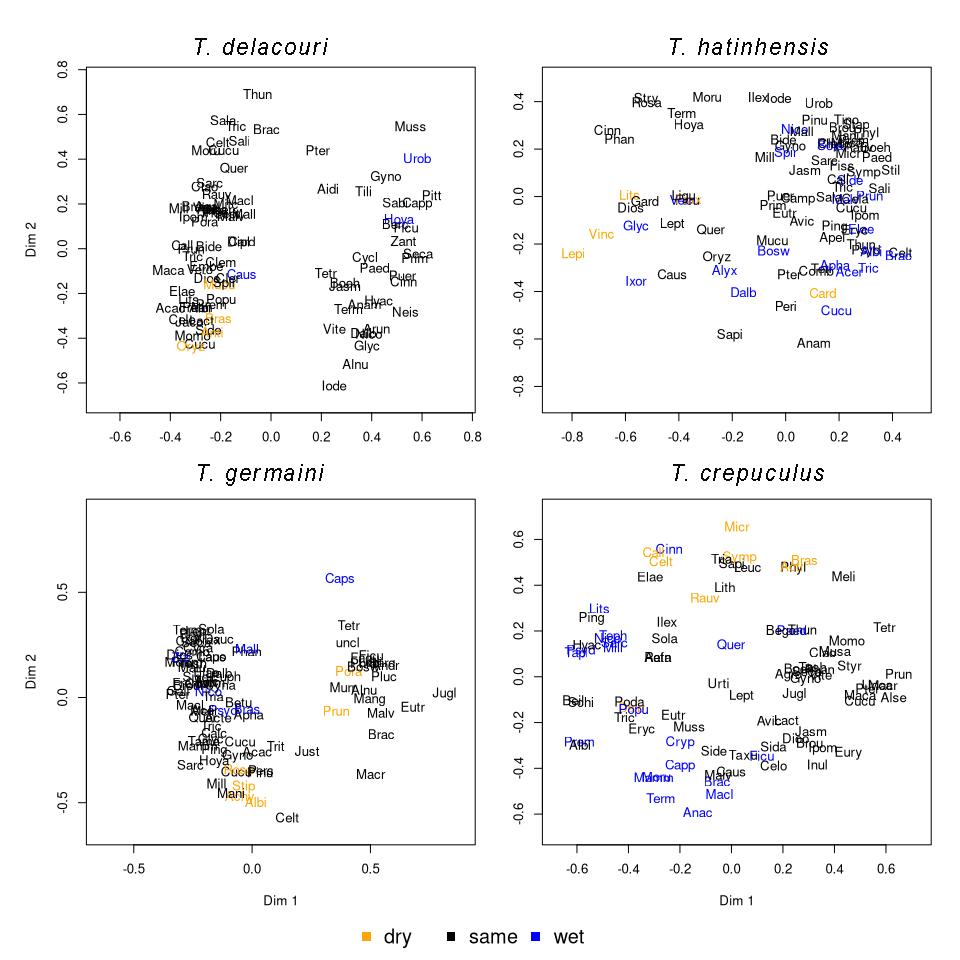


Figure S5. Multidimensional Scaling of the Spearman correlation-based distances (D=(1-rho)/2) of plant families for each langur species. Families where the log2 changes between wet and dry season are lower than -1 (plants found more often in the wet season) are shown in blue, and plant genera found more often in the dry season (log2-foldchange > 1) are shown in orange. Families not having a log2-fold change between -1 and 1 are shown in black. For abbreviations of plant families, see Table S19.


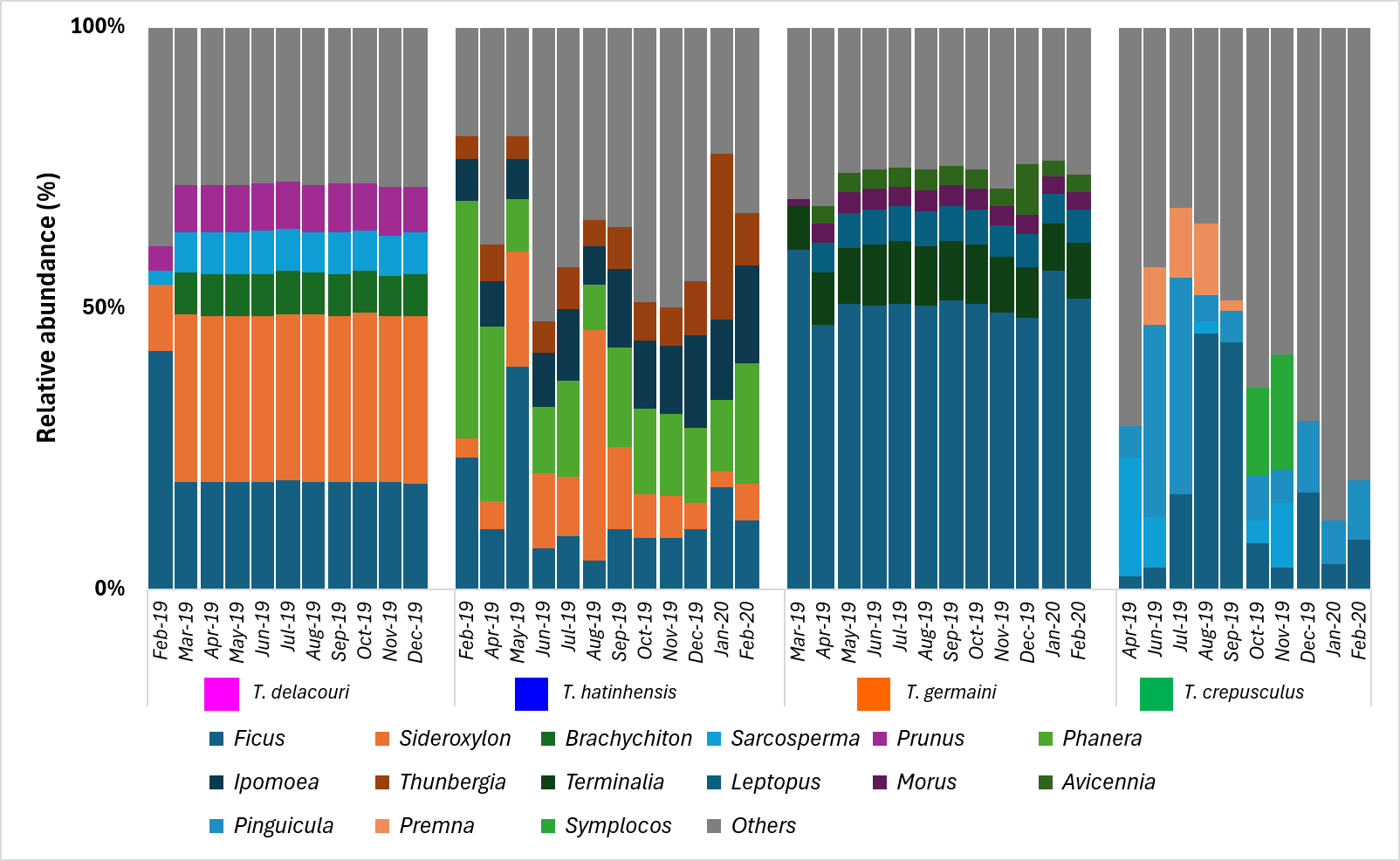


Figure S6. Relative abundance of the top five plant genera among the four langur species studied over a period of 12 consecutive months (note: for T. delacouri and T. crepusculus, data are only available for eleven and ten months, respectively). Stacked bar graphs describe the abundance of genera, with the x-axis representing the langur species in monthly time windows.


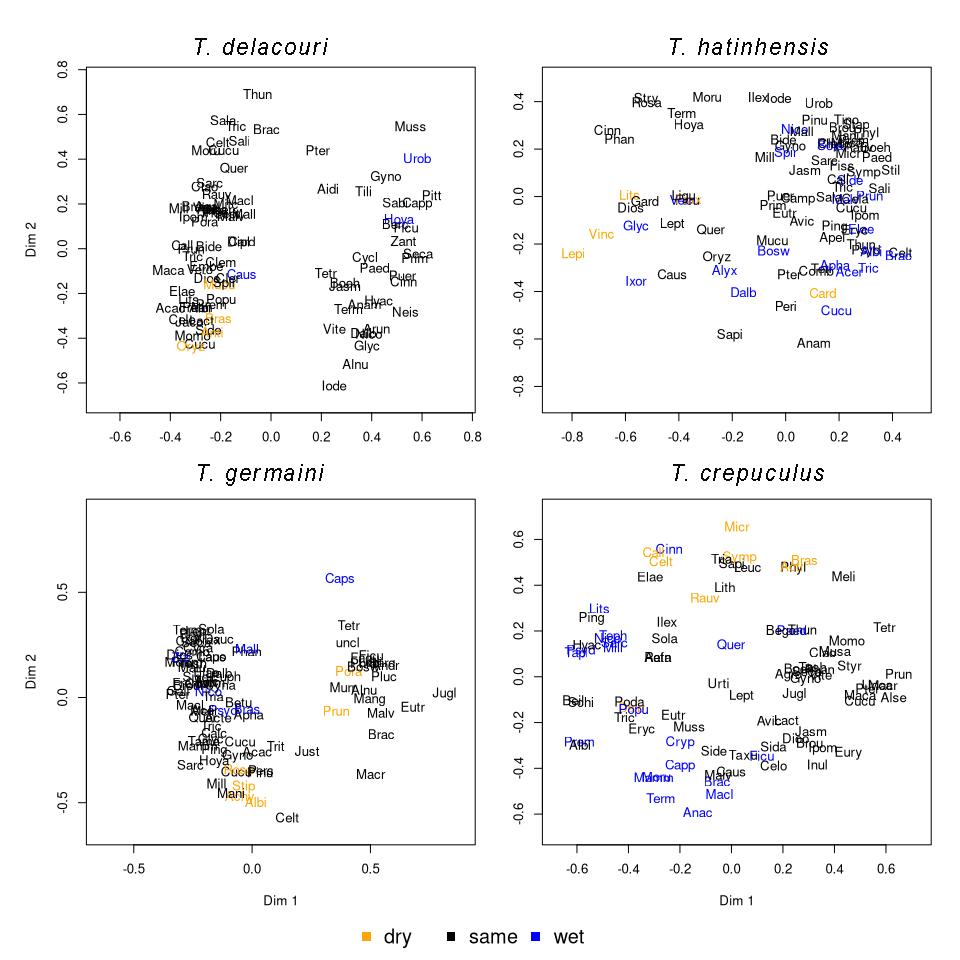


Figure S7. Multidimensional Scaling of the Spearman correlation-based distances (D=(1-rho)/2) of plant genera for each langur species. Genera where the log2 changes between wet and dry season are lower than -1 (plants found more often in the wet season) are shown in blue color, and plant genera found more often in the dry season (log2-fold change > 1) are shown in orange. Plant genera having a log2-fold change between -1 and 1 are shown in black. For abbreviation of plant genera, see Table S20.
